# Induction-Phase rSO_2_–MAP Behaviour and Cross-Clamp Desaturation in NIRS-Guided Selective Carotid Endarterectomy: A Retrospective Cohort Study

**DOI:** 10.3390/jcm15124620

**Published:** 2026-06-14

**Authors:** Ilhan Ozgol, Serkan Ketenciler, Cihan Yucel, Melek Yilmaz, Yasar Gokkurt, Ahmet Ozan Koyuncu, Asime Ay, Mehmet Ali Yesiltas, Cennet Yildiz

**Affiliations:** 1 Department of Cardiovascular Surgery, Prof. Dr. Cemil Tascıoglu City Hospital, Darülaceze Avenue No: 27, Kaptanpaşa District, Şişli, 34384 Istanbul, Türkiye; 2 Department of Anesthesia and Reanimation, Prof. Dr. Cemil Taşçıoğlu City Hospital, Şişli, 34384 Istanbul, Türkiye; 3 Department of Cardiovascular Surgery, ISTUN Sisli Kolan Internatıonal Hospital, 34384 Istanbul, Türkiye; 4 Department of Cardiology, Bakırköy Dr. Sadi Konuk Training and Research Hospital, 34147 Istanbul, Türkiye

**Keywords:** carotid endarterectomy, selective shunting, near-infrared spectroscopy, regional cerebral oxygen saturation, mean arterial pressure, pressure–oxygenation index

## Abstract

**Objective:** The objectives of this study were to characterise induction-phase regional cerebral oxygen saturation (rSO_2_)–mean arterial pressure (MAP) dynamics during near-infrared spectroscopy (NIRS)-guided selective carotid endarterectomy (CEA) and to examine whether the Awake→Intubated pressure–oxygenation pattern may represent an early adjunctive physiological signal of subsequent cross-clamp-related ipsilateral cerebral desaturation. **Methods:** In this retrospective observational cohort study, 322 consecutive elective CEAs managed with an NIRS-guided selective shunting protocol between October 2019 and February 2025 were analysed, after excluding patients considered for routine pre-emptive shunting because of contralateral internal carotid artery occlusion or ≥70% stenosis. Standardised MAP and bilateral rSO_2_ values were extracted at the Awake, Intubated, and Clamp stages, defined as 3 min after carotid cross-clamping. Awake→Intubated ipsilateral ΔrSO_2_/ΔMAP was evaluated as a continuous, exploratory pressure–oxygenation index, with MAP–rSO_2_ directional change classified as concordant or discordant. Clamp-related desaturation was defined as a ≥20% ipsilateral rSO_2_ decrease from Awake to Clamp. Discrimination and adjusted associations were evaluated using receiver operating characteristic analysis and multivariable logistic regression, respectively. **Results:** Clamp-related ≥20% ipsilateral rSO_2_ desaturation occurred in 43 patients (13.4%). The Awake→Intubated ipsilateral ΔrSO_2_/ΔMAP ratio differed significantly between patients with and without ≥20% desaturation and showed significant discrimination on receiver operating characteristic analysis, with an area under the curve (AUC) of 0.799 (95% confidence interval [CI] 0.723–0.876; *p* < 0.001). Concordant pressure–oxygenation change was more frequent among patients with ≥20% desaturation (31/43, 72.1%), whereas discordant change predominated among those without desaturation (228/279, 81.7%; *p* < 0.001). In multivariable analysis, Awake→Intubated ipsilateral ΔrSO_2_/ΔMAP remained associated with clamp-related ≥20% desaturation after adjustment (adjusted odds ratio [OR] 1.63, 95% CI 1.15–2.33; *p* = 0.006), along with symptomatic presentation and 50–69% contralateral stenosis. Postoperative stroke occurred in 4/322 patients (1.2%), and no 30-day mortality occurred. **Conclusions:** During NIRS-guided selective CEA, induction-phase rSO_2_–MAP dynamics were associated with subsequent cross-clamp-related ipsilateral cerebral desaturation. As the outcome was a NIRS-defined desaturation rather than an independent clinical, neurological, or imaging endpoint, these findings indicate association with a surrogate marker rather than prediction of clinically relevant cerebral ischaemia. The Awake→Intubated ΔrSO_2_/ΔMAP ratio and directional pressure–oxygenation pattern may represent early adjunctive physiological signals associated with clamp-related desaturation. These findings are hypothesis-generating and require prospective validation with systematic multimodal monitoring.

## 1. Introduction

Carotid endarterectomy (CEA) reduces long-term ipsilateral stroke risk in patients with clinically significant carotid stenosis, yet perioperative neurological events remain an important concern [1,2]. A major intraoperative challenge is cerebral hypoperfusion during carotid cross-clamping, when antegrade internal carotid flow is interrupted and the adequacy of collateral perfusion varies between patients. An intraluminal shunt may be used routinely in all patients or selectively in those who develop signs of clamp-related hypoperfusion; both strategies have limitations, as unnecessary shunting may increase the risk of thromboembolism or intimal injury, whereas inadequate protection may expose vulnerable patients to hypoperfusion-related ischaemia [1,3]. A selective approach therefore depends on reliable intraoperative identification of patients who do not tolerate cross-clamping, and current guidelines acknowledge the lack of consensus and resulting heterogeneity in monitoring and shunting practice [1].

Near-infrared spectroscopy (NIRS) offers a simple, non-invasive method to monitor regional cerebral oxygen saturation (rSO_2_) continuously and may support intraoperative decision-making alongside established modalities such as stump pressure, electroencephalography/somatosensory evoked potentials, transcranial Doppler ultrasound, or awake neurological assessment [4,5,6]. However, the clinical reliability of fixed absolute rSO_2_ thresholds remains variable across studies, with reported critical decreases differing and no single cut-off achieving consistent diagnostic performance [7]. This suggests that single-time-point rSO_2_ values may incompletely capture a patient’s capacity to maintain cerebral oxygenation when perfusion pressure changes, and that interpreting rSO_2_ together with contemporaneous pressure changes—an approach related to NIRS-based assessment of cerebrovascular autoregulation—may add value [8,9,10].

Physiologically, cerebral blood flow is normally maintained across a range of perfusion pressures by autoregulation, buffering oxygen delivery against fluctuations in systemic arterial pressure [11]. In carotid stenosis, this capacity may be regionally impaired ipsilateral to the lesion, so that cerebral oxygenation becomes more pressure-dependent and may identify patients at higher haemodynamic risk [12]. Combining contemporaneous mean arterial pressure (MAP) changes with the direction of rSO_2_ change may therefore provide a physiologically interpretable adjunct to absolute rSO_2_ triggers. Prospective studies have used NIRS-defined rSO_2_ decreases to detect clamp-related hypoperfusion and to guide selective shunting, supporting the feasibility of this approach while underscoring the variability of the thresholds proposed [13].

In our centre, a bilateral NIRS-guided selective shunting protocol was implemented, and the Awake→Intubated ipsilateral ΔrSO_2_/ΔMAP ratio was retrospectively evaluated as a direction-preserving, exploratory index of induction-phase pressure–oxygenation behaviour. We aimed to characterise induction-phase rSO_2_–MAP dynamics in patients undergoing NIRS-guided selective CEA and to explore whether Awake→Intubated pressure–oxygenation behaviour was associated with subsequent cross-clamp-related ipsilateral cerebral desaturation.

## 2. Methods

### 2.1. Study Design and Population

We retrospectively analysed consecutive patients who underwent elective CEA at our institution between October 2019 and February 2025. Patients were excluded if they underwent concomitant open-heart surgery, had incomplete intraoperative monitoring data, or had contralateral internal carotid artery (ICA) occlusion or ≥70% contralateral stenosis for which routine pre-emptive shunting would be considered. The final cohort comprised 322 patients managed with a NIRS-guided selective shunting protocol.

Patients were eligible for CEA if they had symptomatic 50–99% stenosis or asymptomatic 60–99% stenosis with suitable anatomy and an estimated life expectancy > 5 years [1]. Symptomatic status was defined as ipsilateral stroke, transient ischaemic attack, or amaurosis fugax within 6 months. Demographics, comorbidities, operative variables, and postoperative outcomes were extracted from institutional records.

**Figure 1:**
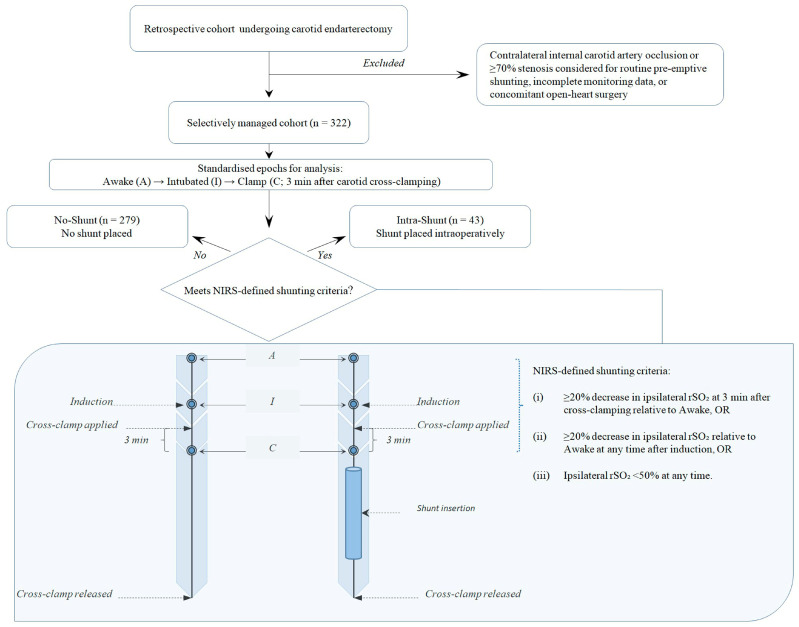
Study flow and near-infrared spectroscopy-guided selective shunting algorithm. Cohort derivation, prespecified intraoperative epochs, and allocation to No-Shunt or Intra-Shunt groups according to NIRS-defined shunting criteria are shown. NIRS = near-infrared spectroscopy; rSO_2_ = regional cerebral oxygen saturation.

Patients were classified according to actual intraoperative shunt use as No-Shunt (no shunt placement; *n* = 279) or Intra-Shunt (shunt placement after NIRS-defined desaturation; *n* = 43). The study flow, cohort derivation, and NIRS-guided shunting algorithm are summarised in Figure 1.

### 2.2. Anaesthesia, Monitoring, and Intraoperative Management

No premedication was given. All procedures were performed under general anaesthesia using a standardised balanced technique: intravenous induction with fentanyl, midazolam, and propofol; rocuronium for tracheal intubation; and sevoflurane maintenance titrated to effect by the attending anaesthesiologist. Patients were ventilated to normocapnia (end-tidal carbon dioxide 35–45 mmHg), with inspired oxygen adjusted to maintain peripheral oxygen saturation ≥95%. Monitoring comprised electrocardiography, pulse oximetry, capnography, invasive arterial pressure, and bilateral near-infrared spectroscopy cerebral oximetry (Masimo Root with O3 Regional Oximetry; Masimo Corporation, Irvine, CA, USA), with sensors on both frontal regions per the manufacturer’s instructions. NIRS and arterial pressure were recorded continuously and interpreted from stable rather than transient readings; technically unreliable values were excluded.

Representative values were extracted at three prespecified time points—Awake (pre-induction baseline), Intubated (induction-phase proxy), and Clamp (3 min after cross-clamping)—with ipsilateral and contralateral rSO_2_ defined by the side of endarterectomy. Mean arterial pressure was kept at or near the Awake baseline throughout; during cross-clamping it was actively managed to prevent a >20% fall (targeting 100–120% of baseline), with ephedrine administered if blood pressure decreased.

### 2.3. Anticoagulation Protocol

Intravenous unfractionated heparin was administered prior to carotid cross-clamping (initial bolus 5000 IU). The activated clotting time (ACT) was remeasured at 3 min, and additional boluses were administered with repeat ACT testing as needed to achieve ACT > 200 s. Carotid cross-clamping was performed only after confirming that the target ACT had been reached.

### 2.4. Shunting Criteria

Selective shunting was guided by intraoperative NIRS monitoring. An intraluminal shunt was placed if any of the following occurred:

A reduction in ipsilateral rSO_2_ ≥20% at 3 min after cross-clamping, relative to Awake;

A reduction in ipsilateral rSO_2_ ≥20% relative to Awake at any time after induction; or

an absolute rSO_2_ < 50% at any time [14,15].

### 2.5. Operative Technique

CEA was performed using standard open techniques. For the standardised NIRS assessment, the common and external carotid arteries (including the superior thyroid artery) were clamped, followed by distal ICA clamping to proceed with endarterectomy. The procedural cross-clamp duration reflects cumulative clamping exposure; in shunted cases it includes shunt insertion/removal steps and the initial test clamp followed by re-clamping and therefore does not represent uninterrupted cerebral ischaemia time. Arteriotomy was closed by primary closure or patch angioplasty based on intraoperative anatomical and technical considerations, including vessel calibre, arteriotomy extent, and distal endpoint quality. All procedures were performed by an experienced vascular surgery team following a standardised institutional protocol.

### 2.6. Postoperative Management and Follow-Up

All patients continued dual antiplatelet therapy (acetylsalicylic acid and clopidogrel) postoperatively, consistent with their preoperative regimen. Patients underwent routine postoperative neurological assessment and standard surveillance. All neurological and systemic complications were recorded during the index hospitalisation and up to 30 days postoperatively through inpatient documentation and outpatient follow-up. Suspected neurological events were evaluated by neurology consultation and confirmed using brain imaging (computed tomography [CT] and/or magnetic resonance imaging [MRI]) as clinically indicated.

### 2.7. Induction-Phase Pressure–Oxygenation Index Assessment

The induction-phase pressure–oxygenation index was derived from signed changes in ipsilateral rSO_2_ and MAP during the Awake→Intubated interval. For each patient, ΔrSO_2_ and ΔMAP were calculated as signed differences between the Intubated and Awake values, i.e., ΔX = X(Intubated) − X(Awake). The ipsilateral ΔrSO_2_/ΔMAP ratio was evaluated as a continuous induction-phase pressure–oxygenation index.

The directional classification relied on the sign of the ΔrSO_2_/ΔMAP ratio rather than its magnitude, with the aim of characterising concordant versus discordant rSO_2_–MAP behaviour rather than deriving a magnitude-based physiological threshold. Concordant pressure–oxygenation change was defined as ΔrSO_2_ and ΔMAP changing in the same direction, whereas discordant pressure–oxygenation change was defined as opposite-direction changes. This descriptive approach is conceptually related to prior NIRS-based assessments of pressure–oxygenation behaviour and cerebrovascular autoregulation [8], but was not treated as a validated measure of cerebral autoregulation.

The induction-phase ipsilateral ΔrSO_2_/ΔMAP ratio and directional pressure–oxygenation pattern were evaluated in relation to clamp-related ipsilateral rSO_2_ desaturation, defined as a ≥20% decrease in ipsilateral rSO_2_ at 3 min after carotid cross-clamping relative to the Awake baseline.

### 2.8. Study Outcomes

The primary analyses had two objectives: (1) to characterise induction-phase pressure–oxygenation behaviour using Awake→Intubated ipsilateral rSO_2_–MAP dynamics and the ΔrSO_2_/ΔMAP index; and (2) to explore the association between induction-phase rSO_2_–MAP behaviour and cross-clamp-related ipsilateral rSO_2_ desaturation, assessed using receiver operating characteristic (ROC) analysis and limited-adjustment logistic regression. Secondary outcomes included perioperative neurological events, intraoperative shunt use, mortality, procedural cross-clamp duration, re-exploration for bleeding or haematoma, cranial nerve injury, and intensive care unit (ICU) and hospital length of stay.

### 2.9. Statistical Analysis

Analyses were performed in IBM SPSS Statistics (version 27; IBM Corp., Armonk, NY, USA). Continuous variables are presented as median (interquartile range [IQR]), and categorical variables as n (%). Normality was assessed with the Shapiro–Wilk test; given non-normal distributions, non-parametric methods were used. Categorical variables were compared using Chi-square or Fisher’s exact tests.

Across the three prespecified time points (Awake, Intubated, and Clamp), rSO_2_ and MAP were analysed using the Friedman test with Bonferroni-adjusted Wilcoxon signed-rank post hoc comparisons. Because three pairwise comparisons were performed, *p* < 0.017 was considered significant for post hoc testing.

Because the Awake value reflects the pre-induction physiological state and corresponds to the institutional NIRS-based shunting trigger, it was used as the primary reference for clamp-related desaturation. To assess whether this reference also captures induction-related changes, a sensitivity analysis repeated the outcome using the Intubated value as reference (≥20% ipsilateral rSO_2_ decrease from Intubated to Clamp).

No patient had a zero ΔMAP denominator, so the ratio was defined for all patients. Because small ΔMAP values can produce disproportionately large ratios, robustness was examined in sensitivity analyses excluding patients with very small denominators (|ΔMAP| ≤ 2 and ≤5 mmHg); the sign-based directional classification was, by construction, independent of ratio magnitude.

The Awake–Intubated ipsilateral ΔrSO_2_/ΔMAP ratio was compared between patients with and without ≥20% ipsilateral rSO_2_ desaturation during cross-clamping using the Mann–Whitney U test. To address potential heterogeneity between symptomatic and asymptomatic patients, the association between the induction-phase ipsilateral ΔrSO_2_/ΔMAP ratio and clamp-related ≥20% desaturation was additionally examined within each subgroup, and a symptomatic-status ×ΔrSO_2_/ΔMAP interaction term was tested in the logistic model. Discrimination was assessed by ROC analysis with area under the curve (AUC). Limited multivariable logistic regression evaluated associations between induction-phase ΔrSO_2_/ΔMAP and clamp-related ≥20% ipsilateral rSO_2_ desaturation. The directional pressure–oxygenation pattern, defined as concordant versus discordant Awake–Intubated changes in ipsilateral rSO_2_ and MAP, was compared according to desaturation status using the Chi-square test.

Model performance was assessed by discrimination (c-statistic), calibration (Hosmer–Lemeshow test), and internal validation by bootstrapping (1000 resamples) to estimate the optimism-corrected c-statistic; the model was regarded as exploratory.

For descriptive purposes, the Youden-optimal operating point of the ratio was reported with sensitivity, specificity, and predictive values; no clinical threshold was proposed. Two-sided *p* < 0.05 was considered statistically significant.

## 3. Results

### 3.1. Study Population

Between October 2019 and February 2025, 322 patients underwent elective CEA under a NIRS-guided selective shunting protocol and were included in the final analysis. Of these, 43 underwent intraoperative shunting after NIRS-defined desaturation, while 279 were managed without shunt placement.

The baseline clinical characteristics are summarised in Table 1. Age, sex, hypertension, current smoking, diabetes mellitus, hyperlipidaemia, coronary artery disease, chronic kidney disease, and left ventricular ejection fraction did not differ significantly between groups. Symptomatic presentation was more frequent in the Intra-Shunt group than in the No-Shunt group (76.7% vs. 54.1%, *p* = 0.005). Contralateral ICA stenosis of 50–69% was also more frequent in the Intra-Shunt group (25.6% vs. 10.8%, *p* = 0.007).

### 3.2. Clinical and Operative Outcomes

The operative and postoperative outcomes are summarised in Table 2. Right-sided CEA and patch closure were numerically more frequent in the Intra-Shunt group, although these differences did not reach statistical significance. Procedural cross-clamp duration was longer in the Intra-Shunt group than in the No-Shunt group (26 [23–27] min vs. 18 [15–22] min, *p* < 0.001). ICU and hospital stays were similar between groups.

Postoperative clinical event rates were low. Postoperative stroke occurred in three patients in the No-Shunt group and one patient in the Intra-Shunt group (1.1% vs. 2.3%, *p* = 0.491). Transient neurological events, re-exploration, and recurrent laryngeal nerve injury did not differ significantly between groups. No 30-day mortality occurred in either group.

### 3.3. Intraoperative Mean Arterial Pressure and Cerebral Oxygenation Across Time Points

Intraoperative MAP and rSO_2_ values across the three prespecified time points are shown in Table 3. In the No-Shunt group, MAP varied significantly across Awake, Intubated, and Clamp time points (*p* < 0.001), decreasing after induction and increasing during cross-clamping. Ipsilateral and contralateral rSO_2_ also varied significantly across time points (both *p* < 0.001). In post hoc analyses, all pairwise comparisons were significant for MAP, ipsilateral rSO_2_, and contralateral rSO_2_.

In the Intra-Shunt group, MAP also varied significantly across time points (*p* < 0.001). Ipsilateral rSO_2_ showed marked temporal variation (*p* < 0.001), with the lowest values observed during clamping. Contralateral rSO_2_ also varied significantly (*p* < 0.001). In post hoc analyses, Intubated versus Clamp MAP did not differ significantly after Bonferroni correction (*p* = 0.062). Awake versus Intubated ipsilateral rSO_2_ (*p* = 0.050) and Awake versus Intubated contralateral rSO_2_ (*p* = 0.789) were also not significant after correction, whereas the remaining pairwise comparisons were significant.

### 3.4. Induction-Phase Pressure–Oxygenation Behaviour and Clamp-Related Desaturation

Clamp-related ≥20% ipsilateral rSO_2_ desaturation occurred in 43 patients (13.4%). The Awake→Intubated ipsilateral ΔrSO_2_/ΔMAP ratio differed significantly between patients with and without clamp-related ≥20% desaturation (Mann–Whitney U = 2406.0, Z = −6.323, *p* < 0.001). Awake→Intubated ipsilateral ΔrSO_2_ also differed between groups (U = 2758.5, Z = −5.723, *p* < 0.001), whereas Awake→Intubated ΔMAP did not (U = 5911.5, Z = −0.153, *p* = 0.878). The Awake→Intubated ΔMAP was comparable between groups (no desaturation: median −14 mmHg [IQR −25 to −5]; desaturation: −14 [−27 to −5]), whereas ipsilateral ΔrSO_2_ differed (no desaturation: +3% [1 to 5]; desaturation: −2% [−4 to 2]); no patient had a zero ΔMAP. Consequently, the ipsilateral ΔrSO_2_/ΔMAP ratio was higher in patients who developed clamp-related ≥20% desaturation (median 0.17 [IQR −0.07 to 0.32]) than in those who did not (−0.14 [−0.45 to −0.06]).

**Figure 2:**
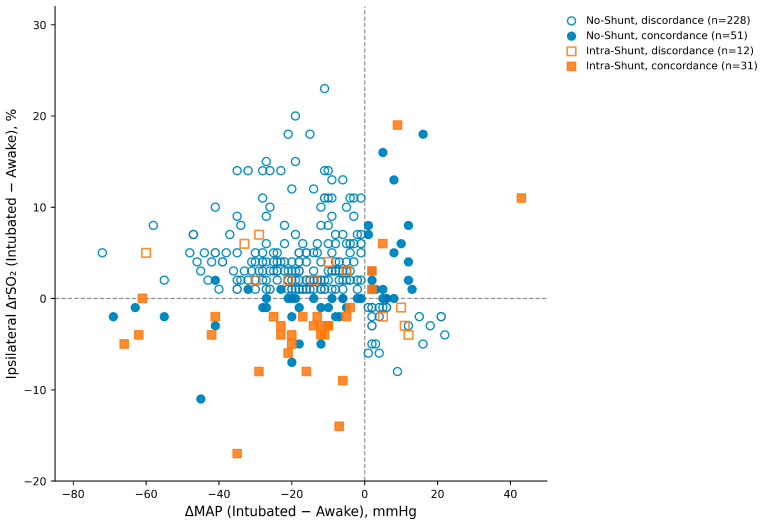
Awake→Intubated pressure–oxygenation pattern according to intraoperative shunt requirement. Scatter plot of individual Awake→Intubated ΔMAP and ipsilateral ΔrSO_2_ values stratified by shunt group and directional pattern. Reference lines at ΔMAP = 0 and ΔrSO_2_ = 0 separate the concordant and discordant quadrants. Directional discordance indicates opposite-direction changes in MAP and ipsilateral rSO_2_, whereas directional concordance indicates same-direction changes; these correspond to negative and zero/positive ΔrSO_2_/ΔMAP ratios, respectively. Δ values were calculated as Intubated minus Awake. MAP = mean arterial pressure; rSO_2_ = regional cerebral oxygen saturation.

During the Awake→Intubated interval, the directional pressure–oxygenation pattern was significantly associated with clamp-related ipsilateral rSO_2_ desaturation status (Table 4). Concordant change was more frequent among patients with ≥20% desaturation (31/43, 72.1%), whereas discordant change predominated among those without ≥20% desaturation (228/279, 81.7%; χ^2^ = 56.844, *p* < 0.001) (Figure 2).

**Figure 3:**
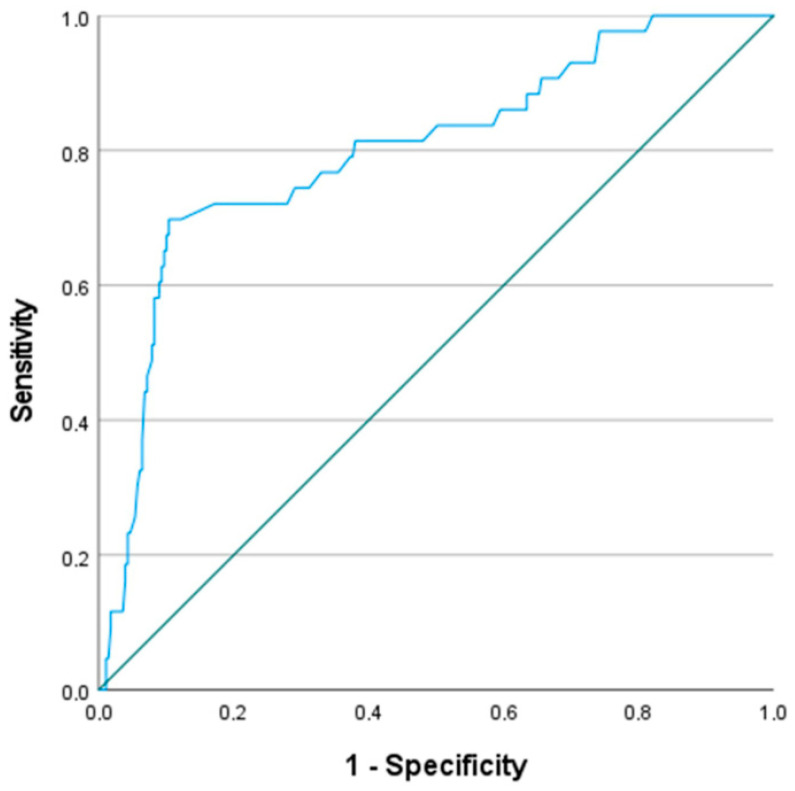
Receiver operating characteristic curve for the Awake→Intubated ipsilateral ΔrSO_2_/ΔMAP ratio in discriminating clamp-related ≥20% ipsilateral rSO_2_ desaturation. The area under the curve was 0.799 (95% confidence interval 0.723–0.876; *p* < 0.001). The blue line represents the ROC curve for the Awake→Intubated ipsilateral ΔrSO_2_/ΔMAP ratio; the diagonal line denotes the reference line of no discrimination (AUC = 0.5). AUC = area under the curve; MAP = mean arterial pressure; rSO_2_ = regional cerebral oxygen saturation; Δ = change from Awake to Intubated.

On ROC analysis, the Awake→Intubated ipsilateral ΔrSO_2_/ΔMAP ratio showed significant discrimination for clamp-related ≥20% ipsilateral rSO_2_ desaturation, with an AUC of 0.799 (95% CI 0.723–0.876; *p* < 0.001) (Figure 3).

At the Youden-optimal operating point (ratio ≈ 0.05), sensitivity was 69.8%, specificity 89.6%, positive predictive value 50.8%, and negative predictive value 95.1% at the observed 13.4% prevalence. This data-derived operating point essentially coincided with zero—the boundary between discordant (negative) and concordant (zero/positive) patterns—consistent with the direction-based interpretation. These operating characteristics are exploratory and in-sample; no ΔrSO_2_/ΔMAP threshold is proposed for clinical decision-making, which would require prospective validation.

In multivariable logistic regression, the Awake→Intubated ipsilateral ΔrSO_2_/ΔMAP ratio remained associated with clamp-related ≥20% ipsilateral rSO_2_ desaturation after adjustment (adjusted odds ratio [OR] 1.63, 95% confidence interval [CI] 1.15–2.33; *p* = 0.006). Symptomatic presentation (adjusted OR 2.24, 95% CI 1.02–4.93; *p* = 0.046) and a contralateral ICA stenosis of 50–69% (adjusted OR 2.39, 95% CI 1.05–5.43; *p* = 0.037) were also associated with desaturation after adjustment (Table 5). The model statistics were: Omnibus χ^2^ = 21.862, *p* < 0.001; Nagelkerke R^2^ = 0.121.

Internal validation by bootstrapping (1000 resamples) showed minimal optimism (optimism-corrected c-statistic 0.762; apparent 0.775). The Hosmer–Lemeshow test indicated suboptimal calibration (*p* < 0.001), driven by extreme ΔrSO_2_/ΔMAP values arising from very small ΔMAP denominators; calibration was acceptable after excluding these cases (|ΔMAP| ≤ 5 mmHg; *p* = 0.49). The model is therefore presented as exploratory and not intended for individual risk prediction.

Clamp-related ≥20% ipsilateral desaturation was more frequent in symptomatic than in asymptomatic patients (33/184 [17.9%] vs. 10/138 [7.2%]; *p* = 0.005). When analysed separately, the Awake→Intubated ipsilateral ΔrSO_2_/ΔMAP ratio was higher in patients who developed desaturation in both subgroups (asymptomatic: 0.11 [IQR −0.12 to 0.24] vs. −0.17 [−0.54 to −0.05], *p* = 0.007; symptomatic: 0.21 [−0.07 to 0.33] vs. −0.13 [−0.38 to −0.06], *p* < 0.001), with comparable discrimination (asymptomatic AUC 0.758 [95% CI 0.56–0.94]; symptomatic AUC 0.806 [95% CI 0.71–0.89]). The symptomatic-status ×ΔrSO_2_/ΔMAP interaction was not significant (adjusted OR 1.45, 95% CI 0.71–2.93; *p* = 0.307), indicating that the association between induction-phase pressure–oxygenation behaviour and clamp-related desaturation did not differ significantly by symptomatic status.

A small number of patients with very small |ΔMAP| produced disproportionately large ratios (20 patients with |ratio| ≥ 2, all with |ΔMAP| ≤ 5 mmHg). Excluding these did not materially change the findings: discrimination was preserved after excluding |ΔMAP| ≤ 2 mmHg (n = 296; AUC 0.793; *p* < 0.001) and |ΔMAP| ≤ 5 mmHg (n = 264; AUC 0.804; *p* < 0.001), and the sign-based directional classification, which does not depend on ratio magnitude, remained significantly associated with desaturation (*p* < 0.001).

In the Intubated-reference sensitivity analysis, ≥20% ipsilateral rSO_2_ desaturation was again observed in 43 patients (13.4%). However, patient-level classification differed in 38 patients (11.8%), with moderate agreement between the Awake-reference and Intubated-reference definitions (κ = 0.490, *p* < 0.001; McNemar *p* = 1.000). The Awake→Intubated ipsilateral ΔrSO_2_/ΔMAP ratio did not differ significantly between patients with and without Intubated-reference desaturation (median −0.143 [IQR −0.333 to 0.250] vs. −0.125 [IQR −0.385 to −0.036]; Mann–Whitney U = 5489.0, *p* = 0.370) and showed no significant discrimination on ROC analysis (AUC 0.542, 95% CI 0.444–0.641; *p* = 0.370). The directional pattern showed the same reference dependence, being unassociated with Intubated-referenced desaturation (χ^2^ = 2.32, *p* = 0.130).

## 4. Discussion

Cerebral perfusion is normally buffered against fluctuations in systemic pressure, yet this buffering capacity may be challenged during the induction phase, carotid manipulation, and cross-clamping. In this setting, continuous bilateral NIRS-derived regional rSO_2_, interpreted alongside MAP, can provide a pragmatic physiological view of pressure–oxygenation behaviour and may complement—rather than replace—established monitoring modalities used for selective shunting during CEA [8,9]. Across the cohort, MAP decreased at the Intubated epoch and increased during clamping, consistent with the haemodynamic effects of anaesthetic agents and subsequent intraoperative adjustments intended to support cerebral perfusion [16,17,18]. Because analyses used prespecified epoch values, interpretation should focus on these time points rather than on continuous trend behaviour outside the sampled epochs.

Against this physiological background, the Awake→Intubated ipsilateral ΔrSO_2_/ΔMAP ratio was evaluated as a direction-preserving, exploratory pressure–oxygenation index rather than as a validated direct measure of cerebral autoregulation. The sign of this ratio was used to characterise concordant versus discordant rSO_2_–MAP behaviour, rather than to derive a magnitude-based physiological threshold. While causality cannot be inferred in this retrospective design, a concordant pressure–oxygenation pattern during induction was more frequent among patients who subsequently developed clamp-related NIRS-defined desaturation, whereas discordant change predominated among those without desaturation. This early signal, occurring before carotid clamping, may reflect a physiological pattern associated with greater susceptibility to induction-related haemodynamic perturbations and to subsequent clamp-related desaturation.

Consistent with this concept, induction-phase rSO_2_–MAP dynamics were associated with subsequent cross-clamp-related ipsilateral rSO_2_ desaturation in the NIRS-guided selective cohort. The Awake→Intubated ipsilateral ΔrSO_2_/ΔMAP ratio showed discriminatory value and remained associated with clamp-related desaturation in multivariable analysis, supporting its potential role as an exploratory adjunctive physiological signal. These analyses concern exploratory physiological characterisation of clamp-related desaturation rather than shunting efficacy or clinical outcome prediction. Therefore, this metric should not be interpreted as an independent trigger for shunt placement, but rather as a hypothesis-generating adjunct that may provide interpretative context alongside absolute rSO_2_ thresholds. Conceptually, this direction-based index may be particularly useful when rSO_2_ changes fall within intermediate “grey-zone” ranges or when absolute changes are difficult to contextualise in isolation.

The induction-phase association was specific to the Awake reference and was not observed when desaturation was referenced to the Intubated value. This dependence on the reference point has two implications. First, it supports the Awake (pre-induction) baseline as the clinically appropriate reference, as it reflects the patient’s true physiological starting point and matches the institutional shunting trigger; an Intubated reference reclassified a substantial proportion of patients and would not have flagged those whose deficit accrued partly during induction. Second, because the induction-phase ratio did not predict the incremental Intubated-to-Clamp change, its association with the Awake-referenced outcome is largely attributable to the shared induction-phase rSO_2_ component. The ratio should therefore be interpreted as an early indicator that the clinically used Awake-referenced threshold is likely to be reached, rather than as an independent predictor of clamp-phase cerebral desaturation.

A concordant induction-phase pattern—ipsilateral rSO_2_ changing in the same direction as MAP—may reflect more pressure-passive behaviour, whereas a discordant pattern may indicate better-preserved oxygenation; these remain cautious physiological interpretations rather than validated autoregulatory measures. Because the direction-based classification depends only on the sign of change, it is not distorted by the disproportionately large ratios arising from very small ΔMAP values and may offer a more stable descriptor than the continuous ratio. Like the continuous ratio, however, this directional signal was apparent only with the Awake reference and not when desaturation was referenced to the Intubated value, suggesting that it largely reflects the shared induction-phase rSO_2_ component.

Several limitations are central to interpretation. First, the retrospective, non-randomised design introduces selection and information/performance bias, as shunt allocation was driven by intraoperative physiology, thereby precluding causal inference. In particular, because intraoperative haemodynamic management responded to the same NIRS signal used to define the outcome, the observed association may reflect intrinsic cerebrovascular vulnerability, the effect of NIRS-responsive clinical management, or both, and these contributions cannot be disentangled in this retrospective design. Second, the relatively small number of patients meeting NIRS-defined desaturation criteria limits the robustness of ROC-based and multivariable analyses; accordingly, the discriminatory findings should be interpreted with caution. The modest explained variance (Nagelkerke R^2^ 0.121) and the calibration findings further indicate that the model should be regarded as exploratory and hypothesis-generating rather than a validated prediction tool. Third, although both symptomatic and asymptomatic patients were included, symptomatic presentation was retained as a covariate and the association between the ΔrSO_2_/ΔMAP ratio and clamp-related desaturation was examined within each subgroup; its direction and magnitude were consistent across subgroups, and the symptomatic-status interaction was non-significant. Nevertheless, the asymptomatic subgroup contained few desaturation events (n = 10), so these stratified analyses are exploratory and warrant prospective confirmation. Fourth, patients with contralateral ICA occlusion or ≥70% contralateral stenosis were excluded because they may undergo pre-emptive routine shunting. As these patients constitute precisely the subgroup at highest risk of clamp intolerance, the present findings apply to a selectively managed, comparatively lower-risk CEA population and should not be extrapolated to patients with severe contralateral disease or a predefined routine-shunt indication. Finally, the direction-based ΔrSO_2_/ΔMAP metric was derived from prespecified interval changes rather than continuous autoregulatory monitoring and should therefore be interpreted as an exploratory pressure–oxygenation index rather than a validated measure of cerebral autoregulation. The absence of direct comparison with other established monitoring approaches further limits external interpretability. Because shunt decisions were based on NIRS-defined criteria without a contemporaneous independent reference standard, the possibility of NIRS-related over- or under-detection of clinically relevant hypoperfusion cannot be quantified. In addition, NIRS-derived rSO_2_ measurements may be influenced by sensor positioning, signal quality, extracranial contamination, patient-specific tissue characteristics, and device-specific algorithms, although technically unreliable readings were excluded from interpretation. Accordingly, these findings should be considered hypothesis-generating with respect to physiological characterisation rather than clinical superiority, and require confirmation in larger prospective studies with systematic multimodal monitoring [1,3,5,6,7].

## 5. Conclusions

Induction-phase rSO_2_–MAP dynamics were associated with subsequent cross-clamp-related ipsilateral cerebral desaturation during NIRS-guided selective carotid endarterectomy. Because this outcome was a NIRS-defined desaturation and no independent reference standard—such as electroencephalography, stump pressure, transcranial Doppler, awake neurological assessment, or postoperative diffusion-weighted imaging—was available, the present analyses describe an association with a surrogate physiological event rather than prediction of clinically relevant cerebral ischaemia or neurological outcome. The Awake→Intubated ΔrSO_2_/ΔMAP ratio and directional pressure–oxygenation pattern may represent early adjunctive physiological signals associated with clamp-related desaturation. Prospective studies with systematic multimodal monitoring are required to validate these findings before clinical implementation.

## Figures and Tables

**Table 1:** Baseline clinical characteristics of the selectively managed cohort according to intraoperative shunt requirement (n = 322).

Variable	No-Shunt (n = 279)	Intra-Shunt (n = 43)	*p* Value
Age, y	68 (62–73)	70 (64–73)	0.516
Male sex	195 (69.9)	28 (65.1)	0.528
Hypertension	197 (70.6)	33 (76.7)	0.407
Current smoking	119 (42.7)	14 (32.6)	0.211
Diabetes mellitus	193 (69.2)	30 (69.8)	0.938
Hyperlipidaemia	175 (62.7)	26 (60.5)	0.776
Coronary artery disease	147 (52.7)	22 (51.2)	0.852
Symptomatic presentation	151 (54.1)	33 (76.7)	0.005
Contralateral ICA stenosis 50–69%	30 (10.8)	11 (25.6)	0.007
Chronic kidney disease	42 (15.1)	6 (14.0)	0.850
Left ventricular ejection fraction, %	60 (55–60)	60 (55–60)	0.572

Data are presented as median (interquartile range) or n (%). Continuous variables were compared using the Mann–Whitney U test; categorical variables were compared using the Chi-square test or Fisher’s exact test, as appropriate. ICA = internal carotid artery.

**Table 2:** Intraoperative and postoperative outcomes of the selectively managed cohort according to intraoperative shunt requirement (n = 322).

Variable	No-Shunt (n = 279)	Intra-Shunt (n = 43)	*p* Value
Right-sided carotid endarterectomy	125 (44.8)	26 (60.5)	0.055
Patch closure	33 (11.8)	9 (20.9)	0.099
Procedural cross-clamp duration, min	18 (15–22)	26 (23–27)	<0.001
Intensive care unit stay, days	1 (1–1)	1 (1–1)	0.078
Hospital stay, days	3 (2–3)	3 (2–3)	0.641
Postoperative stroke	3 (1.1)	1 (2.3)	0.491
Transient neurological event	4 (1.4)	0 (0.0)	0.429
Re-exploration for bleeding/haematoma	14 (5.0)	2 (4.7)	0.918
Recurrent laryngeal nerve injury	1 (0.4)	0 (0.0)	0.694
30-day mortality	0 (0.0)	0 (0.0)	—

Data are presented as median (interquartile range) or n (%). Continuous variables were compared using the Mann–Whitney U test. Categorical variables were compared using the Chi-square test or Fisher’s exact test, as appropriate. No *p* value was calculated for 30-day mortality because no events occurred in either group. Procedural cross-clamp duration reflects cumulative clamping exposure; in shunted cases it includes shunt insertion and removal and the initial test clamp followed by re-clamping and therefore does not represent uninterrupted cerebral ischaemia time.

**Table 3:** Intraoperative changes in mean arterial pressure (MAP) and regional cerebral oxygen saturation (rSO_2_) across three prespecified time points in the selectively managed cohort.

Variable	Awake (A)	Intubated (I)	Clamp (C)	Friedman *p* Value
No-Shunt group (n = 279)				
MAP, mmHg	100 (90–110)	86 (76–94)	96 (90–100)	<0.001
Ipsilateral rSO_2_, %	61 (56–68)	65 (59–72)	59 (54–64)	<0.001
Contralateral rSO_2_, %	64 (58–68)	66 (61–73)	61 (56–67)	<0.001
Intra-Shunt group (n = 43)				
MAP, mmHg	108 (92–120)	90 (82–100)	93 (86–100)	<0.001
Ipsilateral rSO_2_, %	64 (58–68)	63 (56–69)	48 (43–53)	<0.001
Contralateral rSO_2_, %	65 (59–71)	66 (59–71)	60 (52–65)	<0.001

Data are presented as medians (interquartile ranges). Overall time effects were tested with the Friedman test. Pairwise comparisons used Bonferroni-adjusted Wilcoxon signed-rank tests, with *p* < 0.017 considered significant. All pairwise comparisons were significant except in the Intra-Shunt group for MAP Intubated–Clamp (*p* = 0.062), ipsilateral rSO_2_ Awake–Intubated (*p* = 0.050), and contralateral rSO_2_ Awake–Intubated (*p* = 0.789). Ipsilateral and contralateral denote operated and non-operated hemispheres. Clamp values were recorded 3 min after cross-clamping. MAP = mean arterial pressure; rSO_2_ = regional cerebral oxygen saturation.

**Table 4:** Directional pressure–oxygenation pattern during the Awake→Intubated interval according to clamp-related ipsilateral rSO_2_ desaturation status (n = 322).

Direction Pattern	<20% Drop (n = 279)	≥20% Drop (n = 43)	*p* Value
Concordant	51 (18.3)	31 (72.1)	<0.001
Discordant	228 (81.7)	12 (27.9)	

Data are presented as n (% within desaturation group). Concordant pressure–oxygenation change indicates that Awake→Intubated ΔMAP and ipsilateral ΔrSO_2_ changed in the same direction; discordant change indicates opposite-direction change. The *p* value was derived from the Chi-square test. MAP = mean arterial pressure; rSO_2_ = regional cerebral oxygen saturation.

**Table 5:** Multivariable factors associated with cross-clamp-related ipsilateral regional cerebral oxygen saturation (rSO_2_) desaturation (n = 322).

Variable	Adjusted OR (95% CI)	*p* Value
Awake → Intubated ipsilateral ΔrSO_2_/ΔMAP	1.63 (1.15–2.33)	0.006
Symptomatic presentation	2.24 (1.02–4.93)	0.046
50–69% contralateral stenosis	2.39 (1.05–5.43)	0.037

The outcome was a clamp-related ≥20% ipsilateral rSO_2_ decrease from Awake to Clamp. Adjusted odds ratios were derived from multivariable logistic regression. Model fit: Nagelkerke R^2^ = 0.121; Hosmer–Lemeshow *p* < 0.001; optimism-corrected c-statistic 0.762 (1000 bootstrap resamples). The model is exploratory. OR = odds ratio; CI = confidence interval; rSO_2_ = regional cerebral oxygen saturation; ΔMAP = interval-specific change in mean arterial pressure; ΔrSO_2_ = interval-specific change in ipsilateral regional cerebral oxygen saturation.

## Data Availability

The data presented in this study are available on reasonable request from the corresponding author. The data are not publicly available due to privacy and ethical restrictions.
